# Work absenteeism and disability associated with psoriasis and psoriatic arthritis in the USA—a retrospective study of claims data from 2009 TO 2020

**DOI:** 10.1007/s10067-021-05839-9

**Published:** 2021-07-21

**Authors:** A. M. Orbai, S. M. Reddy, N. Dennis, R. Villacorta, S. Peterson, L. Mesana, S. D. Chakravarty, I. Lin, C. S. Karyekar, Y. Wang, M. Pacou, J. Walsh

**Affiliations:** 1 Division of Rheumatology, Psoriatic Arthritis Program, Johns Hopkins University school of Medicine, Baltimore, MD USA; 2 NYU School of Medicine, New York, NY USA; 3 Amaris, Paris, France; 4 Janssen Immunology Global Commercial Strategy Organization, Horsham, PA USA; 5 Amaris, New York, NY USA; 6 Janssen Scientific Affairs, LLC, Horsham, PA USA; 7 Drexel University College of Medicine, Philadelphia, PA USA; 8 Janssen Global Services LLC, Horsham, PA USA; 9 Janssen R&D, LLC, Titusville, NJ USA; 10 University of Utah School of Medicine, George E. Wahlen Veteran Affairs Medical Center, Salt Lake City, UT USA

**Keywords:** Psoriasis, Psoriatic arthritis, Short-term disability, Work absenteeism

## Abstract

**Objectives:**

To compare work absenteeism and short-term disability among adults with psoriasis or psoriatic arthritis (PsA), versus controls in the USA.

**Methods:**

Adults eligible for work absenteeism and/or short-term disability benefits between 1/1/2009 and 4/30/2020 were screened in the IBM® MarketScan® Commercial and Health and Productivity Management Databases. The following groups were defined: (1) psoriasis: ≥ 2 psoriasis diagnoses ≥ 30 days apart and no PsA diagnoses; (2) PsA: ≥ 2 PsA diagnoses ≥ 30 days apart; (3) control: absence of psoriasis and PsA diagnoses. Controls were matched to psoriasis and PsA patients based on age, gender, index year, and comorbidities. Non-recreational work absences and sick leaves were evaluated in absentee-eligible patients, and short-term disability was evaluated in short-term disability-eligible patients. Costs (in 2019 USD) associated with each type of work absence were evaluated.

**Results:**

4261 psoriasis and 616 PsA absentee-eligible and 25,213 psoriasis and 3480 PsA short-term disability-eligible patients were matched to controls. Average non-recreational work absence costs were $1681, $1657, and $1217 for the PsA, psoriasis, and control group, respectively. Compared with psoriasis patients and controls, more PsA patients had sick leaves after 1 year (56.2% versus 55.6% and 41.5%, *p* < 0.0001). Similarly, short-term disability was more frequent in PsA patients than psoriasis patients and controls at year one (8.8% versus 5.6% and 4.7%, *p* < 0.0001) and corresponding costs were higher ($605, $406, and $335 on average, *p* < 0.0001).

**Conclusion:**

Annual work absenteeism and short-term disability were consistently greater among patients with PsA and psoriasis than controls, highlighting the substantial economic burden of psoriatic disease.

**Table:** 

**Key points** *• Patients with PsA had greater short-term disability compared with patients with psoriasis and patients with neither psoriasis nor PsA.* *• Patients with PsA and patients with psoriasis incurred greater non-recreational work absences and sick leaves than patients with neither psoriasis nor PsA.*

**Supplementary information:**

The online version contains supplementary material available at 10.1007/s10067-021-05839-9.

## Introduction

Psoriasis is a chronic inflammatory skin disorder that is characterized by scaly erythematous skin lesions, resulting in itching, irritation, and stinging for most patients [1]. Psoriasis can profoundly affect patient quality of life (QoL) and can follow a relapsing course with patients fluctuating between periods of remission and inflammation. According to data from the National Health and Nutrition Examination Survey 2010, the prevalence of psoriasis in US adults was estimated to be 3.2% (~ 7.2 million cases) [2]. It can affect all ages and prevalence rates between male and female individuals are similar. Family history as well as environmental factors, such as smoking, infections, stress, and certain medications can contribute to disease susceptibility [3, 4]. Psoriasis is also considered a multi-system disease with significant comorbidities; individuals with psoriasis are at increased risk of developing cerebrovascular disease, cardiovascular disorders, diabetes, metabolic syndrome, and depression [1, 5–7].

Psoriatic arthritis (PsA) occurs in up to 30–33% of patients with psoriasis, though it is often underdiagnosed and undertreated [8–10]. PsA is a complex, heterogenous immune-mediated disease characterized by musculoskeletal features, namely, peripheral arthritis, axial disease, enthesitis, and dactylitis. While most patients with PsA have concurrent or prior history of psoriasis, rheumatological manifestations precede the onset of skin lesions in about 20% of patients [11]. During this time, these patients have only a PsA diagnosis (sine psoriasis). PsA can be an aggressive, debilitating disorder that may lead to impaired function, reduced QoL, and irreversible joint damage. The cause of PsA is unknown, but genetic factors as well as environmental factors, such as smoking, stress, and trauma play a role in the susceptibility of developing PsA [12, 13]. In patients with psoriasis, studies have shown that the severity and location of psoriasis, as well as early age of psoriasis onset can also be risk factors for developing PsA [14]. Other risk factors for PsA have been described in patients with psoriasis, such as obesity, infections or genetic history [15]. In addition, the literature describes that patients with PsA have a higher risk of cardiovascular disease [16].

Work disability constitutes a spectrum of states, usually caused by chronic illness, that ranges from reduced productivity while remaining at work, absence from work as sick days (absenteeism), extended work absence with reduced salary or periods of unemployment with receipt of disability benefit (disability leave), to complete cessation of paid employment. Such work disability contributes substantially to the economic burden of psoriasis and PsA. According to a study by the National Psoriasis Foundation (NPF), 49% of people with psoriasis and/or PsA miss work regularly due to illness [7]. Generally, it is estimated that around 20–40% of the total costs of psoriasis are attributable to productivity and work loss [17–22]. Among patients with PsA, it has been found that work disability rates range from 23 to 39% [23, 24]. Additionally, a study that used an employment disadvantages questionnaire to compare patients with psoriasis and PsA found an increased rate of work problems caused by PsA (32% versus 11% for psoriasis; *p* < 0.0005) [25]. Similarly, productivity loss has been shown to be greater among patients with PsA compared with those with other inflammatory diseases such as psoriasis, ankylosing spondylitis, rheumatoid arthritis [22, 26].

To date, previous studies that have analyzed work absence (absenteeism and short-term disability leave) have typically looked only at patients with psoriasis or PsA, rather than comparing the two [17, 18, 21, 27–30]. Fowler et al. conducted a matched analysis between patients with and without psoriasis and found that absenteeism accounts for 40% of the economic burden among patients with psoriasis [21]. When comparing the rates of absenteeism among differing inflammatory diseases, absenteeism was most common among patients with PsA (20.3% versus 14.9%, 8.4% and 10.8% for psoriasis, RA, and AS, respectively) [26]. Data from the NPF survey found that patients with severe psoriasis have 1.8 times greater odds of being unemployed compared with patients with mild psoriasis [7]. Increased severity of psoriasis is also linked to higher costs [19].

Thus far, studies that have analyzed absenteeism are largely based on hospital data or clinical registries [17, 19, 27, 29]. However, there is an unmet need for an up-to-date comparative analysis of absenteeism and short-term disability leave between patients with psoriasis, PsA, and patients without psoriasis and PsA. We used a large administrative claims database to obtain robust estimates among US patients with the most recent data on treatments and standard-of-care.

## Methods

### Data source

This study used the IBM MarketScan® Commercial Claims and Encounters Database (CCAE, January 1, 2009–April 30, 2020), which was linked to the Health and Productivity Management (HPM) Database (January 1, 2009–February 28, 2019). The CCAE Database includes adjudicated health insurance claims (e.g., inpatient, outpatient, and outpatient pharmacy) as well as enrollment data from large employers and health plans who provide private healthcare coverage to employees, their spouses, and dependents. The database contains information on clinical utilization, expenditures, insurance enrollment/plan benefit, inpatient and outpatient information, as well as prescription information. The HPM Database contains workplace absenteeism, short-term disability, long-term disability, and workers’ compensation from a subset of approximately 70 employers included in the CCAE. The HPM data are provided from employer payroll systems and disability case records supplied by data contributors to the CCAE. The use of the IBM MarketScan® databases was reviewed by the New England Institution Review Board (IRB) and was determined to be exempt from broad IRB approval, as this research project did not involve human subject research.

### Sample selection

Patients with at least two diagnoses at least 30 days apart [31] for psoriasis (ICD-9-CM 696.1, ICD-10-CM L40.0-L40.4, L40.8, L40.9) or PsA (ICD-9-CM 696.0, ICD-10-CM L40.5x) were selected from the MarketScan CCAE Database to make up the case groups of psoriasis and PsA. Patients selected in the psoriasis group had no PsA diagnosis in their entire claim records. The index date was defined as the first date of psoriasis or PsA diagnosis.

Patients with no psoriasis or PsA diagnoses in their entire claim records were selected as the control group. The index date was assigned as 12 months after the beginning of continuous enrollment in the database. The control patients were matched 3:1 to cases (psoriasis and PsA combined) based on age, sex, year of the index date, and the number of non-rheumatological comorbidities, identified using the conditions in the Charlson comorbidity index (CCI) [32] at baseline.

All patients assigned to either the case or control group had to be ≥ 18 years old at the index date, have ≥ 12 months of continuous enrollment prior to and after the index date, and be eligible for absenteeism and/or short-term disability benefits for ≥ 12 months after the index date. Patients were considered eligible for absenteeism and/or short-term disability if their employers and/or the database contributors provided such data, although the covered patients may or may not actually incur absence and/or short-term disability in a given time period. Patients with diagnoses for rheumatoid arthritis, ankylosing spondylitis, Crohn’s disease, or ulcerative colitis during the pre-index or follow-up period were excluded. Patients were followed until occurrence of one of the following: inpatient death, end of continuous enrollment or eligibility for absenteeism or short-term disability benefits, or end of study. Death could only be measured using inpatient claims because outpatient deaths are not captured in the CCAE Database.

Several sensitivity analyses were conducted on the patient selection process to test the robustness of the results to the assumptions made. First, different approaches for matching on comorbidities were explored, taking into account more and less strict matching criteria. In one analysis, patients were matched on the full CCI rather than the number of non-rheumatological comorbidities to take into account rheumatic diseases as well as the weights on more severe comorbid conditions. In another, patients were matched on non-psoriasis/PsA-related comorbidities, excluding conditions from the CCI that have been shown to be associated with psoriasis/PsA [33, 34] (rheumatic diseases, myocardial infarction, peripheral vascular disease, cerebrovascular disease, liver disease, and diabetes). Lastly, patients with diagnoses for rheumatoid arthritis, ankylosing spondylitis, Crohn’s disease, or ulcerative colitis during the pre-index period only were excluded.

### Definition of key variables and outcomes

Patient characteristics included key demographic variables (age, sex, and geographic region), insurance plan type, employment status, industry, number of non-rheumatological comorbidities, and the length of follow-up in years.

The study outcomes were related to both absenteeism and short-term disability and were reported per patient per year (PPPY) for up to 5 years of follow-up. As short-term disability coverage is applied after work-leave benefit runs out, there was no overlap between them.

Non-recreational work absences were defined as absences for reasons other than recreational time off (including sick, disability, leave, family medical leave act, or other). Related outcomes included patients with at least one non-recreational work absence each year, the number of work days missed annually due to non-recreational work absences, and the associated costs. As the HPM Database reports the number of hours missed for work absenteeism, 8 h were assumed for an average work day in calculating the number of days missed. The costs were calculated by multiplying the number of hours absent for non-recreational time off by the average hourly wage in the year at which the absence occurred (expressed in 2019 US dollars). Wages were based on the Bureau of Labor Statistics average hourly earnings of all employees on private payrolls, seasonally adjusted [35]. The average hourly wages for each year are reported in the Supplementary Material. Work absences are usually paid in full to employees for brief absences from work. All costs were expressed in 2019 US dollars, adjusted using the Consumer Price Index—All Urban Consumers (CPI-U) [36].

Sick leaves were defined as work absences taken for incidental illness, which is a subcategory of non-recreational work absences. Outcomes related to sick leaves included the proportion of patients with at least one sick leave each year, the number of work days missed annually due to sick leaves (assuming 8 h in an average work day), and the associated costs. The costs related to sick leaves were calculated using the same approach as costs related to other non-recreational work absences.

Short-term disability plans provide benefits for illness or accidents unrelated to work in order to provide partial pay for longer absences, generally for up to 6 to 12 months [37]. Short-term disability outcomes included the percent of patients with at least one short-term disability claim, the number of work days missed due to short-term disability leave, and annual costs associated with short-term disability. The HPM Database reports the number of days missed for short-term disability leaves. Therefore, to calculate costs, the number of days missed was multiplied by eight (average number of hours in a work day) and then multiplied by 0.6 times the average hourly wage (in 2019 US dollars). A factor of 0.6 was applied to the total costs, as this is the median fixed proportion of annual wages paid to private sector employees by short-term disability plans [38, 39].

### Statistical analyses

Descriptive statistics were reported for patient characteristics, absenteeism outcomes, and short-term disability outcomes, where frequency counts and percentages were reported for categorical variables, and mean, standard deviation, median, minimum, maximum, as well as 25th and 75th percentiles were reported for continuous variables. Differences between groups were assessed using Chi-squared tests for categorical outcomes and analysis of variance (ANOVA) for continuous outcomes.

Propensity scores were constructed to create a matched cohort with a pre-defined ratio of 3:1 for the control group and patients with psoriasis and/or PsA. A logistic regression of psoriasis and/or PsA versus the control group as the dependent variable was fit with the following predictors: age, sex, year of the index date, and the number of non-rheumatological comorbidities from the CCI [32] at baseline. The propensity score of each patient was therefore their conditional probability of being in each group.

The costs associated with non-recreational absenteeism and short-term disability were modeled using linear mixed models. A random intercept, slope, or both a random intercept and slope were included and chosen based on the lowest Akaike information criterion (AIC) and Bayesian information criterion (BIC). The odds of having at least one non-recreational work absence or short-term disability leave were modeled using generalized linear mixed models with a logit link and included both a random intercept and slope. Each model was adjusted for insurance plan type and industry to reduce residual confounding after matching and account for the baseline differences in these variables between the groups.

## Results

### Patient characteristics

**Fig. 1:**
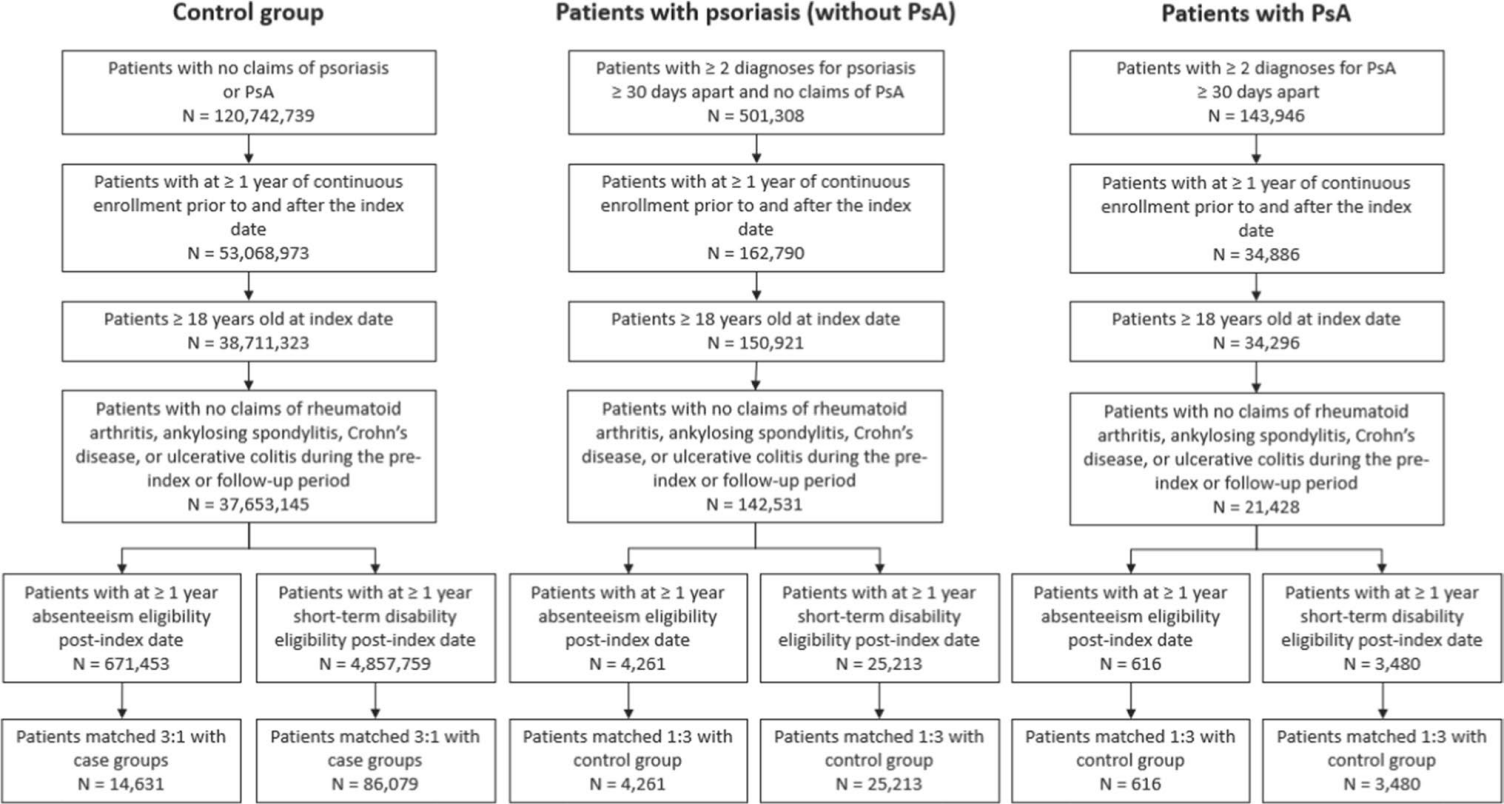
Flow chart of the identification of included patients

The total number of absentee-eligible patients included 14,631 in the control group, 4261 with psoriasis, and 616 with PsA (Fig. 1). The mean age among absentee-eligible patients was 46.9 years for control group, 46.8 years for patients with psoriasis, and 47.5 years for patients with PsA. The majority of patients were male, making up 71.4%, 70.9%, and 76.0% of the control, psoriasis, and PsA groups, respectively. The average length of follow-up was similar among the three groups, ranging from 3.4 to 3.7 years (Table 1).

**Table 1:** Baseline characteristics for the matched populations

	Absentee-eligible patients			Short-term disability-eligible patients		
	Control group	Psoriasis	PsA	Control group	Psoriasis	PsA
Number of patients	14,631	4261	616	86,079	25,213	3480
Age						
Mean (SD)	46.9 (10.0)	46.8 (10.1)	47.5 (9.2)	45.3 (10.0)	45.2 (10.1)	46.3 (9.3)
Median	49	49	49	46	46	47
Min–max	40–55	39–55	41–55	37–54	37–54	39–54
Q1–Q3	21–64	21–64	23–63	19–64	19–64	22–64
Male, *N* (%)	10,441 (71.4%)	3019 (70.9%)	468 (76.0%)	52,200 (60.6%)	15,180 (60.2%)	2215 (63.7%)
Number of comorbidities						
Mean (SD)	0.25 (0.55)	0.26 (0.57)	0.26 (0.58)	0.27 (0.59)	0.26 (0.58)	0.35 (0.68)
Median	0	0	0	0	0	0
Q1–Q3	0–0	0–0	0–0	0–0	0–0	0–1
Min–max	0–6	0–5	0–5	0–7	0–7	0–5
Length of follow-up (years)						
Mean (SD)	3.7 (2.4)	3.7 (2.2)	3.4 (2.1)	3.1 (2.2)	3.2 (2.0)	2.9 (1.9)
Median	3	3	3	3	3	2
Q1–Q3	2–5	2–5	2–5	1–4	2–4	1–4
Min–max	1–9	1–8	1–8	1–9	1–8	1–8
Employment status^*^						
Active full time	12,572 (85.9%)	3960 (92.9%)	582 (94.5%)	84,266 (97.9%)	24,712 (98.0%)	3385 (97.3%)

^*^Non-full-time employees are less likely to be eligible for work absenteeism and disability benefits. However, all patients with eligibility for absenteeism and/or short-term disability benefits for at least 12 months after the index date were included, regardless of their employment status at baseline

A total of 86,079 patients with eligibility for short-term disability were included in the control group, 25,213 in the psoriasis group, and 3480 in the PsA group (Fig. 1). The mean age among short-term disability-eligible patients was 45.3 years for the control group, 45.2 years for patients with psoriasis, and 46.3 years for patients with PsA. Male patients made up 60.6%, 60.2, and 63.7% of the control, psoriasis, and PsA groups, respectively (Table 1).

Between 43 and 58% of patients had a preferred provider organization (PPO) plan, and more than 85% were active full-time employees in every group (Supplementary Material). The most highly represented industry was manufacturing of durable goods, accounting for 39% of absentee-eligible patients and 25% of short-term disability-eligible patients.

### Absenteeism and short-term disability

During the first year of follow-up, 67.7% of patients with PsA and 69.3% of patients with psoriasis had at least one non-recreational work absence, compared with 53.6% of patients in the control group (*p* < 0.0001) (Table 2). Sick leaves were also more common among patients with PsA (56.2%) and patients with psoriasis (55.6%) than the control group (41.5%) in the first year of follow-up (*p* < 0.0001).

**Table 2:** Work absenteeism and short-term disability during follow-up

	Control group	Psoriasis	PsA	*P* value^*^
Number of absentee-eligible patients	14,631	4261	616	
Patients with work absence during 1st follow-up year, *N* (%)				
Non-recreational	7835 (53.6%)	2951 (69.3%)	417 (67.7%)	< 0.0001
Sick leaves	6071 (41.5%)	2368 (55.6%)	346 (56.2%)	< 0.0001
Absenteeism days during follow-up, mean (SD)				
Non-recreational (PPPY)	5.67 (12.97)	7.71 (15.31)	7.81 (13.27)	< 0.0001
Sick leaves (PPPY)	3.17 (7.45)	4.38 (8.70)	4.52 (7.16)	< 0.0001
Costs from hours missed from work, mean (SD)				
Non-recreational (PPPY)	1217.40 (2791.94)	1657.06 (3298.75)	1680.71 (2851.05)	< 0.0001
Sick leaves (PPPY)	682.06 (1606.91)	940.77 (1877.07)	974.27 (1537.99)	< 0.0001
Number of short-term disability-eligible patients	86,079	25,213	3480	
Patients with short-term disability during 1st follow-up year, *N* (%)	4072 (4.7%)	1423 (5.6%)	306 (8.8%)	< 0.0001
Short-term disability days during follow-up, mean (SD)	2.60 (16.94)	3.15 (20.40)	4.69 (24.94)	< 0.0001
Costs associated with short-term disability (PPPY), mean (SD)	334.82 (2184.55)	405.79 (2625.76)	605.36 (3215.77)	< 0.0001

^*^The *p* value corresponds to differences observed among the three groups (controls, psoriasis, and PsA)*. P-*values were calculated using an ANOVA test for continuous variables and a Chi-squared test for categorical variables

More patients with PsA had a short-term disability leave (8.8%) than patients with psoriasis (5.6%) and the control group (4.7%) in the first follow-up year (*p* < 0.0001) (Table 2). The odds of a non-recreational work absence, sick leave, and short-term disability were significantly greater among patients with PsA and patients with psoriasis than the control group during each follow-up year (Table 3). Patients with PsA also had significantly greater odds of short-term disability than patients with psoriasis during the first four years of follow-up.

**Table 3:** Odds ratios (OR) of having at least one non-recreational work absence, sick leave, or short-term disability leave at each follow-up year

	PsA vs. psoriasis	PsA vs. control group	Psoriasis vs. control group
At least one non-recreational work absence, OR [95% CI]			
Year 1	0.93 [0.78, 1.10]	1.67 [1.42, 1.96]	1.80 [1.69, 1.93]
Year 2	1.03 [0.91, 1.16]	1.76 [1.57, 1.97]	1.71 [1.63, 1.80]
Year 3	1.14 [1.00, 1.30]	1.85 [1.64, 2.10]	1.62 [1.55, 1.70]
Year 4	1.27 [1.05, 1.52]	1.95 [1.63, 2.33]	1.54 [1.44, 1.65]
Year 5	1.41 [1.08, 1.82]	2.06 [1.60, 2.64]	1.46 [1.33, 1.61]
At least one sick leave, OR [95% CI]			
Year 1	0.96 [0.82, 1.13]	1.55 [1.33, 1.81]	1.62 [1.52, 1.73]
Year 2	1.01 [0.90, 1.14]	1.58 [1.42, 1.77]	1.56 [1.49, 1.64]
Year 3	1.07 [0.95, 1.20]	1.61 [1.44, 1.81]	1.51 [1.45, 1.58]
Year 4	1.12 [0.95, 1.33]	1.64 [1.40, 1.94]	1.46 [1.37, 1.56]
Year 5	1.19 [0.93, 1.51]	1.68 [1.33, 2.11]	1.41 [1.29, 1.55]
At least one short-term disability leave, OR [95% CI]			
Year 1	1.46 [1.30, 1.64]	1.63 [1.46, 1.82]	1.12 [1.06, 1.18]
Year 2	1.39 [1.28, 1.51]	1.54 [1.42, 1.67]	1.11 [1.07, 1.15]
Year 3	1.32 [1.20, 1.45]	1.46 [1.33, 1.60]	1.10 [1.06, 1.15]
Year 4	1.25 [1.09, 1.44]	1.37 [1.20, 1.57]	1.10 [1.04, 1.16]
Year 5	1.19 [0.98, 1.45]	1.30 [1.08, 1.57]	1.09 [1.01, 1.18]

**Fig. 2:**
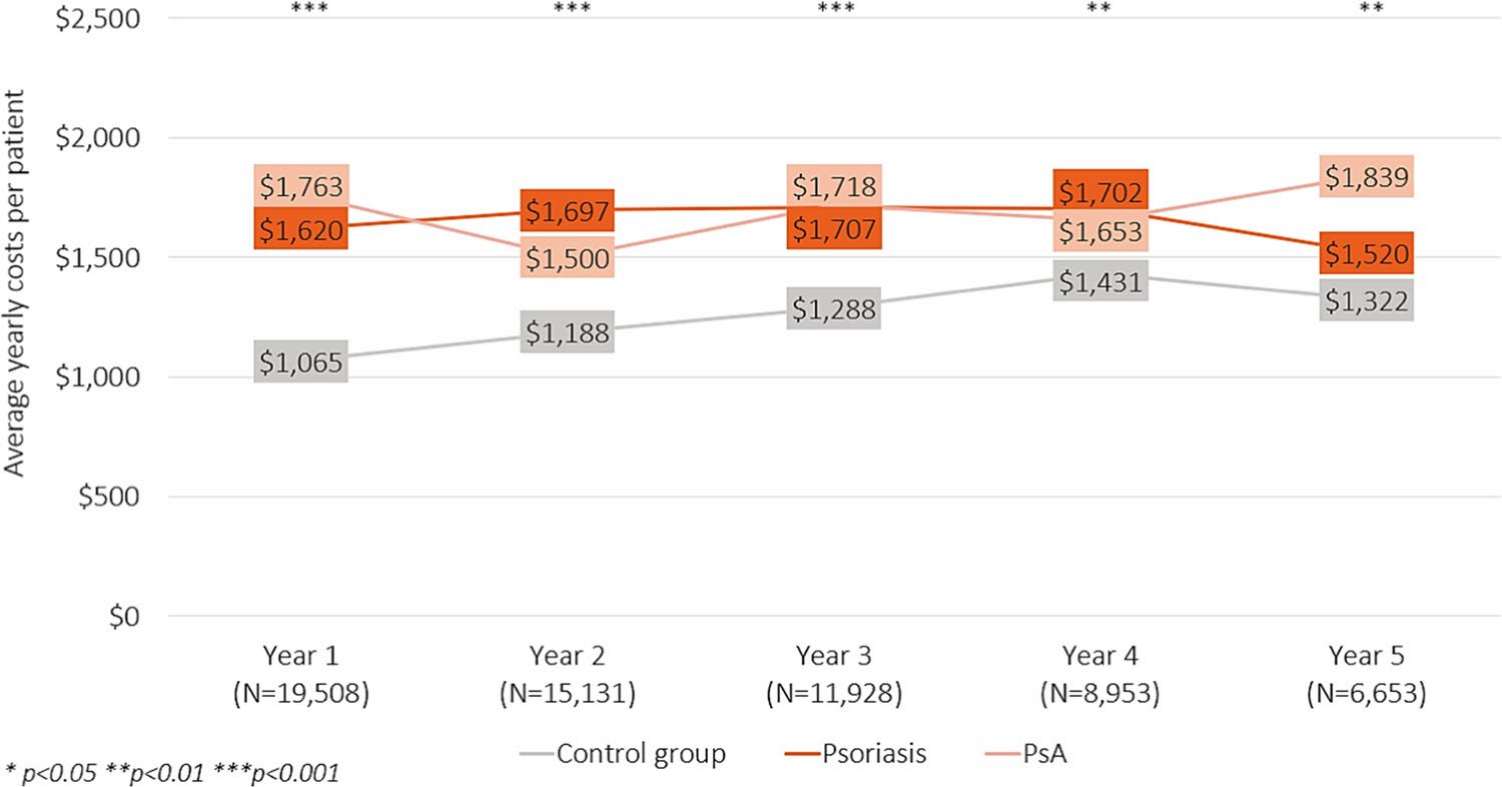
Average costs from non-recreational hours missed from work per patient per year. *P* values were calculated using an ANOVA test and correspond to differences observed among the three groups (controls, psoriasis, and PsA). *P*-values for all pairwise comparisons can be found in the Supplementary Material

**Fig. 3:**
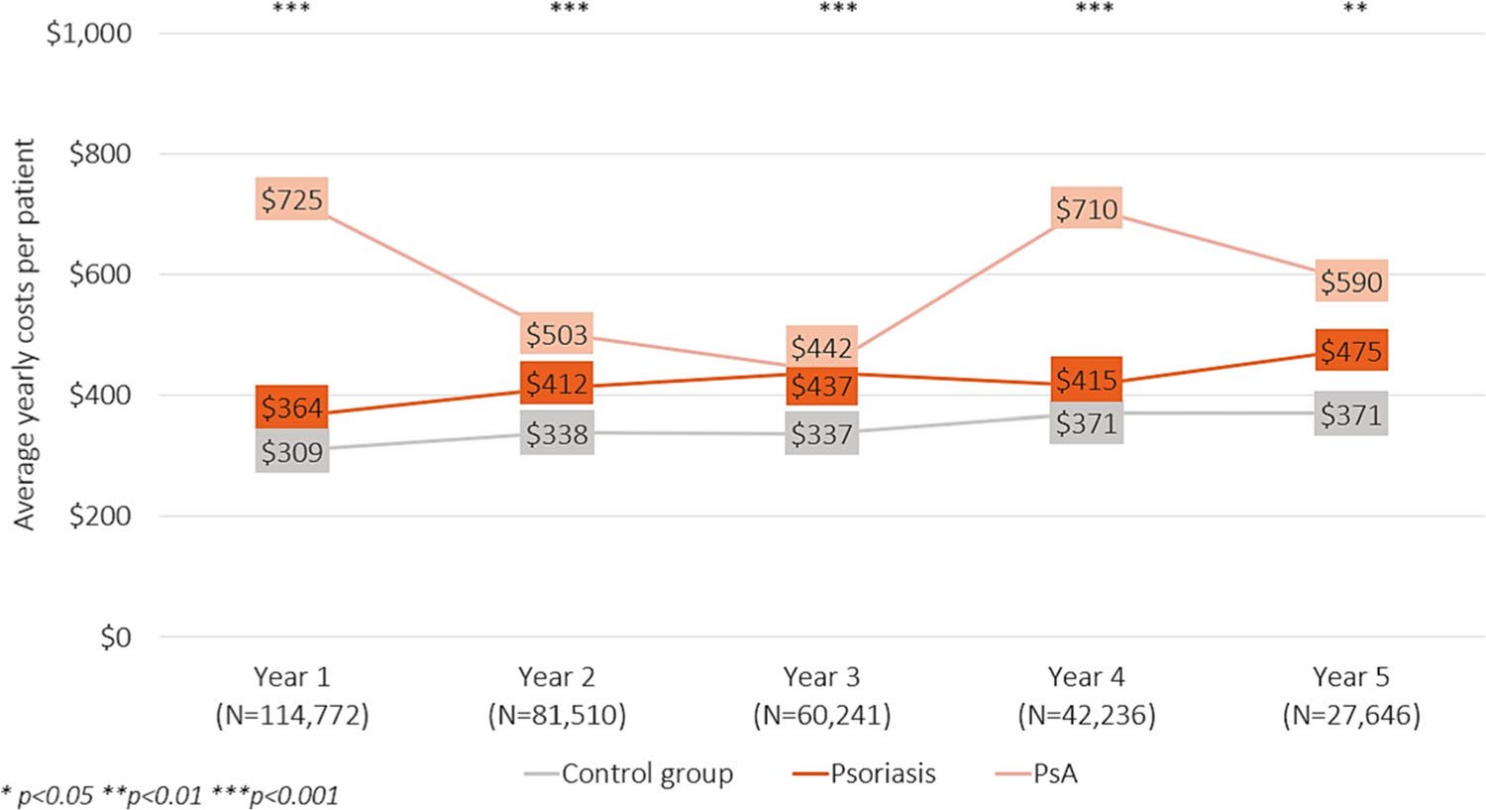
*Average costs associated with short-term disability leave per patient per year. P-*values were calculated using an ANOVA test and correspond to differences observed among the three groups (controls, psoriasis, and PsA). *P*-values for all pairwise comparisons can be found in the Supplementary Material

The costs from non-recreational work absences were on average $1681, $1657, and $1217 PPPY for patients with PsA, patients with psoriasis, and the control group, respectively (Table 2). This trend of increased costs for patients with PsA and psoriasis compared with the control group was sustained throughout 5 years of follow-up (Fig. 2). The costs associated with sick leaves were also greatest among patients with PsA and lowest among the control group (control: $682 PPPY, psoriasis: $941 PPPY, PsA: $974 PPPY). The costs associated with short-term disability were $605, $406, and $335 for patients with PsA, patients with psoriasis, and the control group, respectively (Table 2). This trend of increased costs for patients with PsA compared with the other groups was sustained throughout 5 years of follow-up (Fig. 3). The costs associated with non-recreational work absences and short-term disability were significantly greater among patients with PsA and patients with psoriasis than the control group at year one (*p* < 0.01 for all comparisons) (Supplementary Material). The costs associated with short-term disability were also significantly greater among patients with PsA than psoriasis at year one (*p* < 0.0001).

Sensitivity analyses (using different comorbidity criteria for matching or different criteria for excluding rheumatoid arthritis, ankylosing spondylitis, Crohn’s disease, and ulcerative colitis) showed that non-recreational work absence costs ranged from $1180–$1223 in the control group, $1655–$1657 among patients with psoriasis, and $1681–$1800 among patients with PsA. The short-term disability-related costs ranged from $330–$346 in the control group, $406–$414 among patients with psoriasis, and $605–$644 among patients with PsA.

## Discussion

In this study, evaluations of work absenteeism and short-term disability showed consistently greater associations with PsA and psoriasis than the control group. While work absenteeism outcomes revealed fewer differences between PsA and psoriasis, short-term disability outcomes (the proportion of patients with short-term disability, mean number of days absent, and associated costs) were greater among patients with PsA than patients with psoriasis.

The greater short-term disability incurred by patients with PsA may be linked to greater disease burden and therefore the need for longer work leaves. However, the exact underlying reason for which patients with PsA have greater short-term disability but similar work absenteeism as psoriasis patients cannot be concluded due to the lack of sufficient clinical details from the databases. Little is known from the literature to address this question. Further research is warranted to better understand what is driving work absences and short-term disability leaves among these patient populations.

In a previous study by Fowler et al., analyzing healthcare costs and patient work loss using data from a US-based insurance claims database, mean costs associated with disability per patient per month (PPPM) were $185 for patients with psoriasis and $69 for the control group, and mean costs associated with sick leave PPPM were $43 for patients with psoriasis and $31 for the control group [21]. When extrapolated to yearly values, the corresponding Fowler et al. mean disability costs (PPPY) are considerably higher compared with our findings: $2220 versus $406 for patients with psoriasis and $828 versus $335 for the control group, respectively. However, the mean sick leave costs (PPPY) were lower: $516 versus $941 and $372 versus $974, respectively. The disparities in the disability costs may be due to the assessment of just short-term disability in our study, whereas the Fowler et al. study looked at both short-term and long-term disability claims. Disparities in the sick leave costs may be due to the variations in the calculations: our study used direct sick leave data from employer payroll systems instead of computed sick leaves based on the number of outpatient physician visits (assumed 4 h of sick leave) and inpatient visits (assumed 8 h of sick leave). Furthermore, our study scaled down costs related to short-term disability by 0.6 (typical proportion paid by employers during short-term disability), which was not done in Fowler et al. Finally, our study separately calculated costs related to short-term disability and work absences, whereas Fowler et al. reported a sum of costs. As some patients have data and are eligible for both work absences and short-term disability, the total costs may be underestimated for these patients.

Furthermore, in another previous US-based population study using data from the MarketScan Databases, Feldman et al. found that for patients with psoriasis and PsA, the average adjusted indirect cost from short-term disability was estimated at $1347 PPPY [34]. In contrast, in the present study, the annual short-term disability costs ranged from $406 for patients with psoriasis to $605 for patients with PsA during the 5-year follow-up period. Like the present study, Feldman et al. also assumed an 8-h workday but applied the average hourly wage directly rather than multiplying it by a factor of 0.6 (the average salary proportion paid during a short-term disability leave). This helps explain why greater costs were obtained.

Other arthritic/rheumatic diseases have shown to have a major impact on absenteeism as well. In a retrospective, US-based insurance claims database study of patients with rheumatoid arthritis, short-term disability was associated with adjusted mean annual costs of $466 and corresponding mean sick leave costs were $470 [40]—the latter costs, specifically, are lower than that seen in the present study for both patients with psoriasis and PsA. In a meta-analysis of ankylosing spondylitis studies, annual sick leave costs for patients treated with biologics ranged from €913 to €2336 (approximately equivalent to $1025–$2625) [41]. The greater costs are likely due to the more severe populations, as only patients receiving biologics were considered.

The results of the present study reveal differences in costs associated with work absences among patients with psoriasis and PsA, and sensitivity analyses showed that these findings are robust to the assumptions made.

The study has several limitations. First, biases may arise as a result of the nature of the claims database. While propensity score matching helps reduce bias, certain underlying confounding factors could not be accounted for. For example, disease duration and severity, alcohol, and smoking were not able to be adjusted for given the variables available in the MarketScan Databases [33], which may lead to potential bias or model misspecification. Misclassification may have also occurred given the assumption that patients with psoriasis do not have PsA (as they have no diagnoses in their claims records). The MarketScan HPM Database is a small subset of the Commercial Claims and Encounters Database; male patients and certain employers are more highly represented. In addition, as data were limited to commercially insured patients these findings may not be generalizable to patients who are insured on other plans, uninsured patients, or populations outside of the USA. Furthermore, while this study did not incorporate higher weights associated with more severe comorbidities, matching on the CCI as a sensitivity analysis demonstrated that this assumption did not have a substantial impact on the results. In addition, the control group was matched to patients with psoriasis or PsA, rather than matching separately for each pairwise comparison. As such, the control group more closely resembled the psoriasis group given the greater number of patients with psoriasis than PsA. However, this was the most appropriate approach given the study objectives while limiting the number of matched cohorts. In terms of outcome measurements, long-term disability could not be evaluated due to an insufficient sample size, as less than 0.5% of patients had a long-term disability claim during follow-up. The sample size for absentee-eligible patients with PsA was also small (*n* = 616), which could introduce bias from the higher sampling variability.

In summary, psoriasis and PsA are associated with significantly higher absenteeism and short-term disability, as compared with individuals without psoriasis and PsA. Short-term disability and associated costs are also higher among patients with PsA than patients with psoriasis. These results contribute to our understanding of the economic burden of work loss in employed Americans with psoriasis and PsA. Future studies linking treatment strategies and work absenteeism are needed.

## Supplementary information

Below is the link to the electronic supplementary material.

Supplementary file1 (DOCX 40 KB)
