# Supplementary material for: Work absenteeism and disability associated with psoriasis and psoriatic arthritis in the USA—a retrospective study of claims data from 2009 TO 2020
Source: Clin Rheumatol. 2021 Jul 21;40(12):4933–42. doi: 10.1007/s10067-021-05839-9 (PMC8599387; doi:10.1007/s10067-021-05839-9)
Supplement: Supplementary file 1 — Supplementary file1 (DOCX 40 KB) [file 10067_2021_5839_MOESM1_ESM.docx]

**WORK ABSENTEEISM AND DISABILITY ASSOCIATED WITH PSORIASIS AND PSORIATIC ARTHRITIS IN THE US – A RETROSPECTIVE STUDY OF CLAIMS DATA FROM 2009 TO 2020**

*Clinical Rheumatology*

Orbai AM^1*^, Reddy SM, Dennis N, Villacorta R, Peterson S, Mesana L, Chakravarty SD, Lin I, Karyekar CS, Wang Y, Pacou M, Walsh J

^1^Johns Hopkins Arthritis Center, Baltimore, MD, USA.

*Corresponding author, email: [aorbai1@jhmi.edu](mailto:aorbai1@jhmi.edu)

## Supplementary material

Table 4. ICD-9 and 10 CM codes used for the exclusion of chronic of other inflammatory conditions and to calculate the Charlson Comorbidity Index

| **Condition** | **ICD-9-CM codes** | **ICD-10-CM codes** |
| --- | --- | --- |
| **Chronic inflammatory conditions excluded^[[1]](#footnote-1)^** | | |
| Rheumatoid arthritis | 714.0, 714.1, 714.2, 714.3x | M05.x, M06.x |
| Ankylosing spondylitis | 720.x | M45.x |
| Crohn’s disease | 555.x | K50.x |
| Ulcerative colitis | 556.x | K51.x |
| **Charlson Comorbidity Index^[[2]](#footnote-2)^** | | |
| Myocardial infarction | 410.x, 412.x | I21.x, I22.x, I25.2 |
| Congestive heart failure | 398.91, 402.01, 402.11, 402.91, 404.01, 404.03, 404.11, 404.13, 404.91, 404.93, 425.4 - 425.9, 428.x | I09.9, I11.0, I13.0, I13.2, I25.5, I42.0, I42.5 - I42.9, I43.x, I50.x, P29.0 |
| Peripheral vascular disease | 093.0, 437.3, 440.x, 441.x, 443.1 - 443.9, 447.1, 557.1, 557.9, V43.4 | I70.x, I71.x, I73.1, I73.8, I73.9, I77.1, I79.0, I79.2, K55.1, K55.8, K55.9, Z95.8, Z95.9 |
| Cerebrovascular disease | 362.34, 430.x - 438.x | G45.x, G46.x, H34.0, I60.x - I69.x |
| Dementia | 290.x, 294.1, 331.2 | F00.x - F03.x, F05.1, G30.x, G31.1 |
| Chronic pulmonary disease | 416.8, 416.9, 490.x - 505.x, 506.4, 508.1, 508.8 | I27.8, I27.9, J40.x - J47.x, J60.x - J67.x, J68.4, J70.1, J70.3 |
| Rheumatic disease | 446.5, 710.0 - 710.4, 714.0 - 714.2, 714.8, 725.x | M05.x, M06.x, M31.5, M32.x - M34.x, M35.1, M35.3, M36.0 |
| Peptic ulcer disease | 531.x - 534.x | K25.x - K28.x |
| Mild liver disease | 070.22, 070.23, 070.32, 070.33, 070.44, 070.54, 070.6, 070.9, 570.x, 571.x, 573.3, 573.4, 573.8, 573.9, V42.7 | B18.x, K70.0 - K70.3, K70.9, K71.3 - K71.5, K71.7, K73.x, K74.x, K76.0, K76.2 - K76.4, K76.8, K76.9, Z94.4 |
| Diabetes without complications | 250.0 - 250.3, 250.8, 250.9 | E10.0, E10.1, E10.6, E10.8, E10.9, E11.0, E11.1, E11.6, E11.8, E11.9, E12.0, E12.1, E12.6, E12.8, E12.9, E13.0, E13.1, E13.6, E13.8, E13.9, E14.0, E14.1, E14.6, E14.8, E14.9 |
| Diabetes with complications | 250.4 - 250.7 | E10.2 - E10.5, E10.7, E11.2 - E11.5, E11.7, E12.2 - E12.5, E12.7, E13.2 - E13.5, E13.7, E14.2 - E14.5, E14.7 |
| Paraplegia and hemiplegia | 334.1, 342.x, 343.x, 344.0 - 344.6, 344.9 | G04.1, G11.4, G80.1, G80.2, G81.x, G82.x, G83.0 - G83.4, G83.9 |
| Renal disease | 403.01, 403.11, 403.91, 404.02, 404.03, 404.12, 404.13, 404.92, 404.93, 582.x, 583.0 - 583.7, 585.x, 586.x, 588.0, V42.0, V45.1, V56.x | I12.0, I13.1, N03.2 - N03.7, N05.2 - N05.7, N18.x, N19.x, N25.0, Z49.0 - Z49.2, Z94.0, Z99.2 |
| Cancer | 140.x - 172.x, 174.x - 195.8, 200.x - 208.x, 238.6 | C00.x - C26.x, C30.x - C34.x, C37.x - C41.x, C43.x, C45.x - C58.x, C60.x - C76.x, C81.x - C85.x, C88.x, C90.x - C97.x |
| Moderate/severe liver disease | 456.0 - 456.2, 572.2- 572.8 | I85.0, I85.9, I86.4, I98.2, K70.4, K71.1, K72.1, K72.9, K76.5, K76.6, K76.7 |
| Metastatic carcinoma | 196.x - 199.x | C77.x - C80.x |
| AIDS/HIV | 042.x - 044.x | B20.x - B22.x, B24.x |

Table 5. Additional baseline characteristics for the matched populations

|  | **Absentee-eligible patients** | | | **Short-term disability-eligible patients** | | |
| --- | --- | --- | --- | --- | --- | --- |
|  | **Control group** | **Psoriasis** | **PsA** | **Control group** | **Psoriasis** | **PsA** |
| **Number of patients** | 14,631 | 4,261 | 616 | 86,079 | 25,213 | 3,480 |
| **Insurance plan type - N (%)** |  |  |  |  |  |  |
| Comprehensive | 73 (0.5%) | 14 (0.3%) | 3 (0.5%) | 1944 (2.3%) | 493 (2.0%) | 78 (2.2%) |
| EPO | 145 (1.0%) | 114 (2.7%) | 14 (2.3%) | 911 (1.1%) | 328 (1.3%) | 35 (1.0%) |
| HMO | 1617 (11.1%) | 536 (12.6%) | 75 (12.2%) | 6708 (7.8%) | 2409 (9.6%) | 367 (10.5%) |
| POS | 2591 (17.7%) | 669 (15.7%) | 79 (12.8%) | 4517 (5.2%) | 1674 (6.6%) | 209 (6.0%) |
| PPO | 6386 (43.6%) | 2148 (50.4%) | 318 (51.6%) | 49993 (58.1%) | 14543 (57.7%) | 2011 (57.8%) |
| POS with capitation | 0 (0%) | 0 (0%) | 0 (0%) | 75 (0.1%) | 58 (0.2%) | 7 (0.2%) |
| CDHP | 2881 (19.7%) | 618 (14.5%) | 101 (16.4%) | 10495 (12.2%) | 3466 (13.7%) | 482 (13.9%) |
| HDHP | 795 (5.4%) | 136 (3.2%) | 23 (3.7%) | 10520 (12.2%) | 2078 (8.2%) | 271 (7.8%) |
| Unknown | 143 (1.0%) | 26 (0.6%) | 3 (0.5%) | 916 (1.1%) | 164 (0.7%) | 20 (0.6%) |
| **Geographic region - N (%)** |  |  |  |  |  |  |
| Northeast | 1928 (13.2%) | 629 (14.8%) | 71 (11.5%) | 14415 (16.7%) | 5203 (20.6%) | 583 (16.8%) |
| North Central | 3711 (25.4%) | 943 (22.1%) | 134 (21.8%) | 19176 (22.3%) | 5713 (22.7%) | 784 (22.5%) |
| South | 5544 (37.9%) | 1419 (33.3%) | 224 (36.4%) | 34464 (40.0%) | 9285 (36.8%) | 1379 (39.6%) |
| West | 3428 (23.4%) | 1268 (29.8%) | 185 (30.0%) | 17943 (20.8%) | 4980 (19.8%) | 731 (21.0%) |
| Unknown | 20 (0.1%) | 2 (0.0%) | 2 (0.3%) | 81 (0.1%) | 32 (0.1%) | 3 (0.1%) |
| **Employment status** |  |  |  |  |  |  |
| Active Full Time | 12572 (85.9%) | 3960 (92.9%) | 582 (94.5%) | 84266 (97.9%) | 24712 (98.0%) | 3385 (97.3%) |
| Active Part Time or Seasonal | 1960 (13.4%) | 261 (6.1%) | 28 (4.5%) | 1295 (1.5%) | 329 (1.3%) | 50 (1.4%) |
| Early retiree | 3 (0.0%) | 1 (0.0%) | 0 (0.0%) | 14 (0.0%) | 5 (0.0%) | 0 (0.0%) |
| Retiree (status unknown) | 1 (0.0%) | 0 (0.0%) | 0 (0.0%) | 15 (0.0%) | 0 (0.0%) | 0 (0.0%) |
| COBRA Continuee | 1 (0.0%) | 0 (0.0%) | 0 (0.0%) | 33 (0.0%) | 4 (0.0%) | 0 (0.0%) |
| Long Term Disability | 3 (0.0%) | 0 (0.0%) | 1 (0.2%) | 1 (0.0%) | 4 (0.0%) | 4 (0.1%) |
| Other/Unknown | 91 (0.6%) | 39 (0.9%) | 5 (0.8%) | 455 (0.5%) | 159 (0.6%) | 41 (1.2%) |
| **Industry** |  |  |  |  |  |  |
| Oil & Gas Extraction, Mining | 532 (3.6%) | 249 (5.8%) | 31 (5.0%) | 638 (0.7%) | 113 (0.4%) | 11 (0.3%) |
| Manufacturing, Durable Goods | 5448 (37.2%) | 1946 (45.7%) | 298 (48.4%) | 20814 (24.2%) | 6832 (27.1%) | 987 (28.4%) |
| Manufacturing, Nondurable Goods | 1969 (13.5%) | 338 (7.9%) | 48 (7.8%) | 9121 (10.6%) | 2407 (9.5%) | 351 (10.1%) |
| Transportation, Communications, Utilities | 5321 (36.4%) | 1358 (31.9%) | 195 (31.7%) | 13086 (15.2%) | 4805 (19.1%) | 691 (19.9%) |
| Retail Trade | 0 (0%) | 0 (0%) | 0 (0%) | 6226 (7.2%) | 1329 (5.3%) | 187 (5.4%) |
| Finance, Insurance, Real Estate | 0 (0%) | 0 (0%) | 0 (0%) | 18139 (21.1%) | 5877 (23.3%) | 673 (19.3%) |
| Services | 1361 (9.3%) | 370 (8.7%) | 44 (7.1%) | 16770 (19.5%) | 3616 (14.3%) | 547 (15.7%) |
| Construction | 0 (0%) | 0 (0%) | 0 (0%) | 120 (0.1%) | 35 (0.1%) | 3 (0.1%) |
| Wholesale | 0 (0%) | 0 (0%) | 0 (0%) | 388 (0.5%) | 100 (0.4%) | 14 (0.4%) |
| Unknown | 0 (0%) | 0 (0%) | 0 (0%) | 777 (0.9%) | 99 (0.4%) | 16 (0.5%) |

EPO: Exclusive Provider Organization; HMO: Health Maintenance Organization; POS: Point-of-service; PPO: Preferred Provider Organization; CDHP: Consumer-Driven Health Plan; HDHP: High-Deductible Health Plan; COBRA: Consolidated Omnibus Budget Reconciliation Act

Table 6. Average hourly wages used to calculate the costs associated with work absences and short-term disability, based on the Bureau of Labor Statistics average hourly earnings of all employees on private payrolls, seasonally adjusted, and expressed in 2019 dollars

| **Year** | **Average hourly wage^[[3]](#footnote-3)^** | **CPI-U Inflation (2019=100)^[[4]](#footnote-4)^** | **Average hourly wage**  **(2019 US dollars)** |
| --- | --- | --- | --- |
| 2009 | $22.17 | 83.90 | $26.42 |
| 2010 | $22.58 | 85.30 | $26.47 |
| 2011 | $23.03 | 88.00 | $26.17 |
| 2012 | $23.47 | 89.80 | $26.14 |
| 2013 | $23.96 | 91.10 | $26.30 |
| 2014 | $24.46 | 92.60 | $26.41 |
| 2015 | $25.01 | 92.70 | $26.98 |
| 2016 | $25.65 | 93.90 | $27.32 |
| 2017 | $26.31 | 95.90 | $27.43 |
| 2018 | $27.10 | 98.20 | $27.60 |
| 2019 | $28.00 | 100.00 | $28.00 |

The following formulas were used to calculate the average annual costs associated with non-recreational work absences, sick leaves, and short-term disability.

**Non-recreational work absences:**

$$Costs_{yi}=Hours_{yi}*Wage_{y}*CPI_{y}$$

Where $Costs_{yi}$ are the total costs associated with non-recreational work absences in year $y$ for the $i$th patient, $Hours_{yi}$ are the total number of hours missed by patient $i$ in year $y$ due to non-recreational work absences, $Wage_{y}$ is the average hourly wage in year $y$ (as shown in the table above), and $CPI_{y}$ is the inflation factor using the CPI-U, with the index year being 2009 (as shown in the table above).

**Sick leaves:**

$$Costs_{yi}=Hours_{yi}*Wage_{y}*CPI_{y}$$

Where $Costs_{yi}$ are the total costs associated with sick leaves in year $y$ for the $i$th patient, $Hours_{yi}$ are the total number of hours missed by patient $i$ in year $y$ due to sick leaves, $Wage_{y}$ is the average hourly wage in year $y$ (as shown in the table above), and $CPI_{y}$ is the inflation factor using the CPI-U, with the index year being 2009 (as shown in the table above).

**Short-term disability:**

$$Costs_{yi}=Days_{yi}*8*Wage_{y}*CPI_{y}*0.6$$

Where $Costs_{yi}$ are the total costs associated with short-term disability in year $y$ for the $i$th patient, $Days_{yi}$ are the total number of days missed by patient $i$ in year $y$ due to short-term disability, $Wage_{y}$ is the average hourly wage in year $y$ (as shown in the table above), and $CPI_{y}$ is the inflation factor using the CPI-U, with the index year being 2009 (as shown in the table above).

$Days_{yi}$ is multiplied by 8 to obtain the total number of hours missed due to short-term disability, assuming an average of 8 hours in a workday. The total costs are multiplied by a factor of 0.6 as this is a standard proportion of wages paid during short-term disability leave.

Table 7. Mixed-model results for non-recreational absenteeism, sick leaves, and short-term disability costs

|  |  | **Average cost difference**  **[95% CI]** | **P-value** |
| --- | --- | --- | --- |
| **Costs associated with non-recreational absenteeism** | | |  |
| Difference in costs at 1 year | |  |  |
|  | PsA vs. Control group | $382.68 [$197.78, $567.58] | <0.0001 |
|  | Psoriasis vs. Control group | $398.35 [$320.48, $476.22] | <0.0001 |
|  | PsA vs. Psoriasis | -$15.68 [-$208.98, $177.63] | 0.874 |
| Incremental cost for each additional year of follow up | |  |  |
|  | PsA | -$7.16 [-$106.98, $92.65] | 0.888 |
|  | Psoriasis | -$8.57 [-$44.67, $27.53] | 0.642 |
|  | Control group | $69.65 [$49.43, $89.86] | <0.0001 |
| **Costs associated with sick leaves** | |  |  |
| Difference in costs at 1 year | |  |  |
|  | PsA vs. Control group | $236.97 [$131.95, $341.99] | <0.0001 |
|  | Psoriasis vs. Control group | $210.25 [$166.01, $254.49] | <0.0001 |
|  | PsA vs. Psoriasis | $26.72 [-$83.07, $136.52] | 0.633 |
| Incremental cost for each additional year of follow up | |  |  |
|  | PsA | $21.88 [$-36.25, $80.02] | 0.461 |
|  | Psoriasis | $30.15 [$9.12, $51.18] | 0.005 |
|  | Control group | $58.67 [$46.90, $70.44] | <0.0001 |
| **Costs associated with short-term disability** | | |  |
| Difference in costs at 1 year | |  |  |
|  | PsA vs. Control group | $310.98 [$245.80, $376.16] | <0.0001 |
|  | Psoriasis vs. Control group | $35.07 [$8.28, $61.85] | 0.010 |
|  | PsA vs. Psoriasis | $275.92 [$207.90, $343.94] | <0.0001 |
| Incremental cost for each additional year of follow up | |  |  |
|  | PsA | -$47.25 [-$86.22, -$8.28] | 0.018 |
|  | Psoriasis | $18.26 [$4.64, $31.87] | 0.009 |
|  | Control group | $6.49 [-$0.96, $13.93] | 0.088 |

Table 8. P-values corresponding to all pairwise comparisons presented in Fig. 2 and Fig. 3

|  | **PsA vs. Psoriasis** | **PsA vs.**  **Control group** | **Psoriasis vs. Control group** |
| --- | --- | --- | --- |
| **Costs associated with non-recreational absences - p-value*** | |  |  |
| Year 1 | 0.3325 | <0.0001 | <0.0001 |
| Year 2 | 0.1072 | 0.0035 | <0.0001 |
| Year 3 | 0.9445 | 0.0085 | <0.0001 |
| Year 4 | 0.7682 | 0.0897 | 0.0001 |
| Year 5 | 0.0913 | 0.0072 | 0.0514 |
| **Costs associated with short-term disability - p-value*** | |  |  |
| Year 1 | <0.0001 | <0.0001 | 0.0005 |
| Year 2 | 0.1362 | 0.0043 | <0.0001 |
| Year 3 | 0.9199 | 0.0484 | 0.0003 |
| Year 4 | 0.0245 | 0.0039 | 0.0290 |
| Year 5 | 0.3297 | 0.0457 | 0.0108 |

*P-values were calculated using t-tests. If a significant difference between the variances was observed, then the Welch’s unequal variances t-test was used. Otherwise, a Student’s t-test was used.

1. Hudesman DP, Chakravarty SD, Emond B, Ellis LA, Lefebvre P, Sadik K, Scher JU (2020) Healthcare resource utilization and costs associated with inflammatory bowel disease among patients with chronic inflammatory diseases: a retrospective cohort study. BMC rheumatology 4:1-13 [↑](#footnote-ref-1)
2. Quan H, Sundararajan V, Halfon P, Fong A, Burnand B, Luthi JC, Saunders LD, Beck CA, Feasby TE, Ghali WA. Coding algorithms for defining comorbidities in ICD-9-CM and ICD-10 administrative data. Medical care. 2005 Nov 1:1130-9. [↑](#footnote-ref-2)
3. U.S. Bureau of Labor Statistics Average Hourly Earnings of All Employees: Total Private. https://fred.stlouisfed.org/series/CES0500000003. [↑](#footnote-ref-3)
4. U.S. Bureau of Labor Statistics CPI-All Urban Consumers (Current Series). https://www.bls.gov/cpi/data.htm. [↑](#footnote-ref-4)
